# Novel deep learning-based solution for identification of prognostic subgroups in liver cancer (Hepatocellular carcinoma)

**DOI:** 10.1186/s12859-021-04454-4

**Published:** 2021-11-24

**Authors:** Alice R. Owens, Caitríona E. McInerney, Kevin M. Prise, Darragh G. McArt, Anna Jurek-Loughrey

**Affiliations:** 1 School of Electronics, Electrical Engineering and Computer Science, Queen’s University Belfast, 18 Malone Road, Belfast, BT9 5BN Northern Ireland UK; 2 Patrick G. Johnson Centre for Cancer Research, Queen’s University Belfast, Belfast, Northern Ireland UK

**Keywords:** Hepatocellular carcinoma, Deep learning, Clustering, Prognostic subgroups, Autoencoders, Survival analysis, Liver cancer

## Abstract

**Background:**

Liver cancer (Hepatocellular carcinoma; HCC) prevalence is increasing and with poor clinical outcome expected it means greater understanding of HCC aetiology is urgently required. This study explored a deep learning solution to detect biologically important features that distinguish prognostic subgroups. A novel architecture of an Artificial Neural Network (ANN) trained with a customised objective function (L_RSC_) was developed. The ANN should discover new data representations, to detect patient subgroups that are biologically homogenous (clustering loss) and similar in survival (survival loss) while removing noise from the data (reconstruction loss). The model was applied to TCGA-HCC multi-omics data and benchmarked against baseline models that only use a reconstruction objective function (BCE, MSE) for learning. With the baseline models, the new features are then filtered based on survival information and used for clustering patients. Different variants of the customised objective function, incorporating only reconstruction and clustering losses (L_RC_); and reconstruction and survival losses (L_RS_) were also evaluated. Robust features consistently detected were compared between models and validated in TCGA and LIRI-JP HCC cohorts.

**Results:**

The combined loss (L_RSC_) discovered highly significant prognostic subgroups (*P*-value = 1.55E−77) with more accurate sample assignment (Silhouette scores: 0.59–0.7) compared to baseline models (0.18–0.3). All L_RSC_ bottleneck features (N = 100) were significant for survival, compared to only 11–21 for baseline models. Prognostic subgroups were not explained by disease grade or risk factors. Instead L_RSC_ identified robust features including 377 mRNAs, many of which were novel (61.27%) compared to those identified by the other losses. Some 75 mRNAs were prognostic in TCGA, while 29 were prognostic in LIRI-JP also. L_RSC_ also identified 15 robust miRNAs including two novel (hsa-let-7g; hsa-mir-550a-1) and 328 methylation features with 71% being prognostic. Gene-enrichment and Functional Annotation Analysis identified seven pathways differentiating prognostic clusters.

**Conclusions:**

Combining cluster and survival metrics with the reconstruction objective function facilitated superior prognostic subgroup identification. The hybrid model identified more homogeneous clusters that consequently were more biologically meaningful. The novel and prognostic robust features extracted provide additional information to improve our understanding of a complex disease to help reveal its aetiology. Moreover, the gene features identified may have clinical applications as therapeutic targets.

**Supplementary Information:**

The online version contains supplementary material available at 10.1186/s12859-021-04454-4.

## Background

Hepatocellular carcinoma (HCC) contributes to around 90% of primary liver cancers [1] and is associated with cirrhosis linked to hepatitis B and C infection [2]. In the US, it is one of the fastest growing causes of death from cancer [3]. Thus, the expansion of knowledge on HCC disease aetiology is important. Identifying patient subgroups that stratify by survival due to biological differences will be a step forward towards this goal. This information could in future enable precision medicine whereby patients, when profiled using omics technologies, are stratified into subgroups and have their treatments tailored accordingly. This approach to patient management could improve overall survival in HCC. Moreover, the biological information gained through the identification of prognostic subgroups could facilitate the discovery of new biomarkers and targets for therapies.

Availability of omics data for diseases, including cancers and HCC, is growing exponentially. However, the high dimensionality of this data can make identifying biologically relevant patterns extremely challenging. This scenario necessitates the development of new analytical solutions that harness the power of artificial intelligence (AI) to reveal new information. Deep learning has been explored for patient subgroup identification in different cancers using high-dimensional multi-omics data [4, 5]. Chaudhary et al. applied deep learning in the area of HCC [6] to identify significantly different survival subgroups using autoencoders. Autoencoders are feedforward neural networks which can be used to learn a new representation of data, typically for dimensionality reduction. They encode their input into a latent space and then decode this latent representation as their output. The latent space can be used for further analysis, such as clustering patients into groups and identifying key features. Autoencoders have proven popular in bioinformatics as they can integrate multiple omics and data types [5, 6]. Autoencoder transformation can often aggregate genes by pathway, which is useful for biological interpretation and revealing the underlying patterns [7].

Training an Artificial Neural Network (ANN), such as an autoencoder, is typically an iterative process which uses an objective function, also known as a loss function. The loss function comprises of a mathematical formula that is designed for a specific task that we are trying to train our model for (e.g. clustering). The loss function is used to assess how well the network is performing and to guide the network updates for the next iteration to help arrive at the optimum solution for the task at hand. For an autoencoder, where the goal is reconstruction of the data (i.e. dimensionality reduction), the loss function is used to evaluate how well the original (input) data can be retrieved from the learnt (reduced) data representation (referred to as bottleneck). However, the effectiveness of an autoencoder can be measured in a way more suited to the problem space. If the latent space an autoencoder produces is used to group patients in a survival sensitive way and identify features of interest, then using an objective function that incentivises a latent space with survival and clustering relevance is important. For an ANN to produce features of survival relevance, a supervised approach can be taken, with the inclusion of survival analysis techniques directly into the loss function. The Cox proportional hazards model, a popular survival analysis technique, evaluates variables to assess their impact on an event, usually death. It has been utilised in deep learning solutions to help make survival predictions [8, 9]. In addition to assessing the survival relevance of the latent space in the loss function, the clustering quality of the space can also be examined using methods underpinning common clustering techniques such as k-means. Incorporating a k-means objective into the loss function of a network has been explored singly in order to produce cluster-friendly representations of data [10]. Clustering data can formulate substructure to reveal distinct groups that are biologically homogeneous and consequently meaningful. In precision medicine, biologically distinct patient groups may have clinical relevance for diagnosing patients into disease subtypes and receiving their particular treatments.

This study aimed to explore solutions which take advantage of both survival analysis and clustering techniques when training an autoencoder with multi-omics data. The motivation for considering both metrics (together with the autoencoder reconstruction loss) in the training process was to obtain groups of patients which are distinct in terms of survival and that are biologically insightful. Methods are applied in HCC, a disease that is very heterogeneous and complex due to diverse risk factors. The autoencoder architecture used by Chaudhary et al. [6] for multi-omics data analysis is used as a state-of-the-art baseline. The baseline first trains a standard autoencoder model and then utilises survival information to filter bottleneck features, which are further used for patient clustering. In our work, autoencoder architectures were explored that incorporated survival-based and clustering-based losses directly into the loss function of an autoencoder model. The losses were examined separately and combined together as a hybrid model. Evaluation of the five different models and their cluster quality was performed using the Silhouette score and with survival analysis via the log-rank test statistic. The hybrid model proved to be a superior method as it identified significantly different prognostic groups that were far more homogeneous than those of the baseline models. Prognostic groups were distinguished by a large number of features that were consistently identified by the hybrid model. Many of these robust features were novel compared to the other losses and a proportion could be validated as prognostic in two HCC cohorts, thereby indicating their biological relevance and potential for therapeutic applications as biomarkers or targets. This new information increases our understanding of the aetiology of this heterogeneous disease. Potentially in future it may also improve the clinical diagnosis and treatment of HCC implementing a precision medicine approach.

## Methods

### Datasets

Multi-omics (miRNA, RNA-Seq, methylation) and survival data for primary liver tumour samples of HCC from The Cancer Genome Atlas (TCGA) was analysed. TCGA data was downloaded and pre-processed using TCGA-assembler 2 [11], in an approach following that of Chaudhary et al. [6] For RNA-seq, normalized counts of genes collected using the Illumina HiSeq assay were analysed. For miRNA, data collected using the Illumina HiSeq assay was analysed, with hg19 as the reference genome and miRNA information from mirBase 20. For methylation, data collected using the Infinium HumanMethylation 450 BeadChip assay was selected for analysis. Methylation values were averaged, with 1500 base pairs ahead of transcription start sites being selected to indicate the genomic region for which the average value should be calculated. Only those samples which had all three omics types, a non-negative survival value and a histologic diagnosis of HCC were selected. For each omics type, features which had either a missing or zero value in more than 20% of samples were removed. Next, those samples which had more than 20% of their features missing or of zero value were removed. The impute.knn function in the impute R package was used to fill in any missing values [12]. Following pre-processing, a total of 352 samples were taken forward for further analyses.

The three data types were concatenated into a single vector for each patient creating the multi-omics matrix, which was used as the input for the proposed model. The final dataset consisted of 35,024 features for 352 patients. The Liver Cancer, Riken Japan (LIRI-JP) HCC dataset, which also had associated survival data, was utilised as an independent cohort for feature validation [13]. The data was accessed using the HCCDB platform online (http://lifeome.net/database/hccdb/home.html). Data consisted of gene expression measures also collected using RNA-seq for 212 HCC samples from 203 patients. Both HCC cohorts had similar clinical characteristics (sex ratios, age profiles) and underlying health conditions with risk factors such as hepatitis B and C (Table 1).

**Table 1:** Clinical characteristics and risk factors of the TCGA and LIRI-JP HCC cohorts

TCGA	Gender		
		Male	239
		Female	113
	Grade	G1	52
		G2	165
		G3	120
		G4	11
		Not available	4
	Age at diagnosis	< 18	2
		18–29	10
		30–49	53
		50–69	209
		70+	78
	Risk factors	Alcohol consumption	114
		Hepatitis B	103
		Hepatitis C	53
		Non-alcoholic fatty liver disease	19
		Hemochromatosis	5
		Alpha-1 antitrypsin deficiency	1
		No history of primary risk factors	83
		Other	21
LIRI-JP	Gender	Male	153
		Female	50
	Age	30–49	15
		50–69	94
		70+	94
	Risk factors	Hepatitis B	57
		Hepatitis C	121
		Non-B Non-C	29

A summary of the clinical characteristics and risk factors associated with the TCGA (N = 352) and the LIRI-JP HCC cohorts (N = 203)

### Model construction

As the baseline, the autoencoder of Chaudhary et al. [6] was recreated. Herein, their autoencoder model was implemented and trained using log loss, also known as binary cross entropy (BCE). In addition, mean squared error (MSE), termed L_R_ was used with the same baseline autoencoder architecture as a comparison. The formula of L_R_ is presented in Eq. 1:

1 $$L_{R} = \frac{1}{n}\sum\limits_{i = 1}^{n} {\left\| {x_{i} - \psi \left( {\phi \left( {x_{i} } \right)} \right)} \right\|}^{2}$$

where $x$ represents input, $\phi$ represents the encoder function of the autoencoder, $\psi$ represents the decoder function, meaning $\psi \left( {\phi \left( x \right)} \right)$ represents the final output of the model. These baseline models utilise survival information to filter bottleneck features after network training and then use the selected features for clustering. In our implementation of the baseline, the bottleneck produced by the autoencoder trained with MSE for ten epochs was clustered using the KMeans function from Scikit-learn Python library (full algorithm with kmeans++ initialisation) into *k* groups ranging from 2 to 5. The optimal number of *k* was identified as being two using the Silhouette score, estimated using the silhouette_score function from Scikit-learn Python library. This result was in line with previous findings presented in the baseline work [6].

For the network construction, the Keras [14] module tf.Keras in Tensorflow was used [15]. As before [6], the three omics data types were stacked by sample to form a single matrix, which was unit norm scaled. This was done using the normalize function from the Scikit-learn pre-processing module [16]. The autoencoder, as before [6], was created using hidden layers of dimensions 500, 100 and 500. As before [6] *tanh* was used as the activation function throughout, dropout was set to 0.5, an L2 regularization penalty of 0.0001 was applied to the output and an L1 regularization penalty of 0.001 was applied to the kernel. Stochastic gradient descent was used as the optimizer with the batch size set to one and epochs set to ten.

To explore a cluster-based loss and determine whether it could identify prognostic subgroups in HCC that were biologically distinct, a custom autoencoder was created. For the custom autoencoder construction, hidden layers of dimension 1000, 100 and 1000 were used, with Sigmoid activation throughout. Data from the three omics types was stacked by sample to form a single matrix. The matrix was scaled in the range of 0 to 1 using the MinMaxScaler from Scikit-learn [16]. Batch size was set to sample size (N = 352) and the Adam optimiser selected. An L1 regularization penalty of 0.001 was applied to the kernel to control exploding gradients. The overall clustering loss used L_R_ and L_C_ to form: L_RC_ = αL_R_ + βL_C_ where α and β are the parameters of the model.

The clustering-based loss L_C_ was used to evaluate the quality of cluster produced as an output of the k-means clustering on the bottleneck of the autoencoder. With a k-means clustering algorithm, samples are divided into *k* groups, where *k* is a pre-defined parameter. This is an iterative process where each group is represented by a centroid which is calculated as the mean of the data points (samples) within this group. Samples are assigned to the cluster with the nearest centroid. Following group assignments, centroids are recalculated. Typically, this process continues until group assignments no longer change. The L_C_ was driven from the Silhouette score, commonly used for cluster evaluation. The Silhouette score [17] of a data point $i$ from a cluster $A$ is formulated as per Eq. 2:

2 $$s\left( i \right) = \frac{b\left( i \right) - a\left( i \right)}{{max\left\{ {a\left( i \right), b\left( i \right)} \right\}}}$$

where $a\left( i \right)$ is the mean distance of $i$ to all other data points in $A$ and $b\left( i \right)$ is the smallest mean distance between $i$ and all data points of any other cluster of which *i* is not a member. With our model, the *L*_*C*_ aims to minimise the distance of each sample to its nearest centroid and to maximise the distance to its next closest centroid. The formula of $L_{C}$ is presented in Eq. 3.

3 $$L_{C} = \frac{1}{n}\left\{ {\sum\limits_{i = 1}^{n} {\left\| {\phi \left( {x_{i} } \right) - \mu_{i} } \right\|}^{2} - \sum\limits_{i = 1}^{n} {\left\| {\phi \left( {x_{i} } \right) - \lambda_{i} } \right\|}^{2} } \right\}$$

where $\mu_{i}$ represents the centroid closest to the bottleneck vector of input $x_{i}$ ($\phi \left( {x_{i} } \right)$). Conversely $\lambda_{i}$ represents the next nearest centroid to the bottleneck vector of input $x_{i}$_._

In order to determine the initial centroids and group assignments for losses utilising L_C_, the custom autoencoder was initially run with only the L_R_ loss for one epoch. All data was then passed through the network and the bottleneck layer predicted. The two vectors with the furthest Euclidean distance were selected to be the initial centroids. Each sample was then assigned to the centroid with the shortest squared Euclidean distance to their bottleneck vector. With the seed centroids and group assignments determined, the custom autoencoder was then trained using L_RC_. After each epoch, centroids were updated by taking the mean of each bottleneck feature for the samples in the relevant group, followed by the reassignment of groups as before. For losses using L_C_, samples were not shuffled during training for the purposes of maintaining the group assignments.

**Fig. 1:**
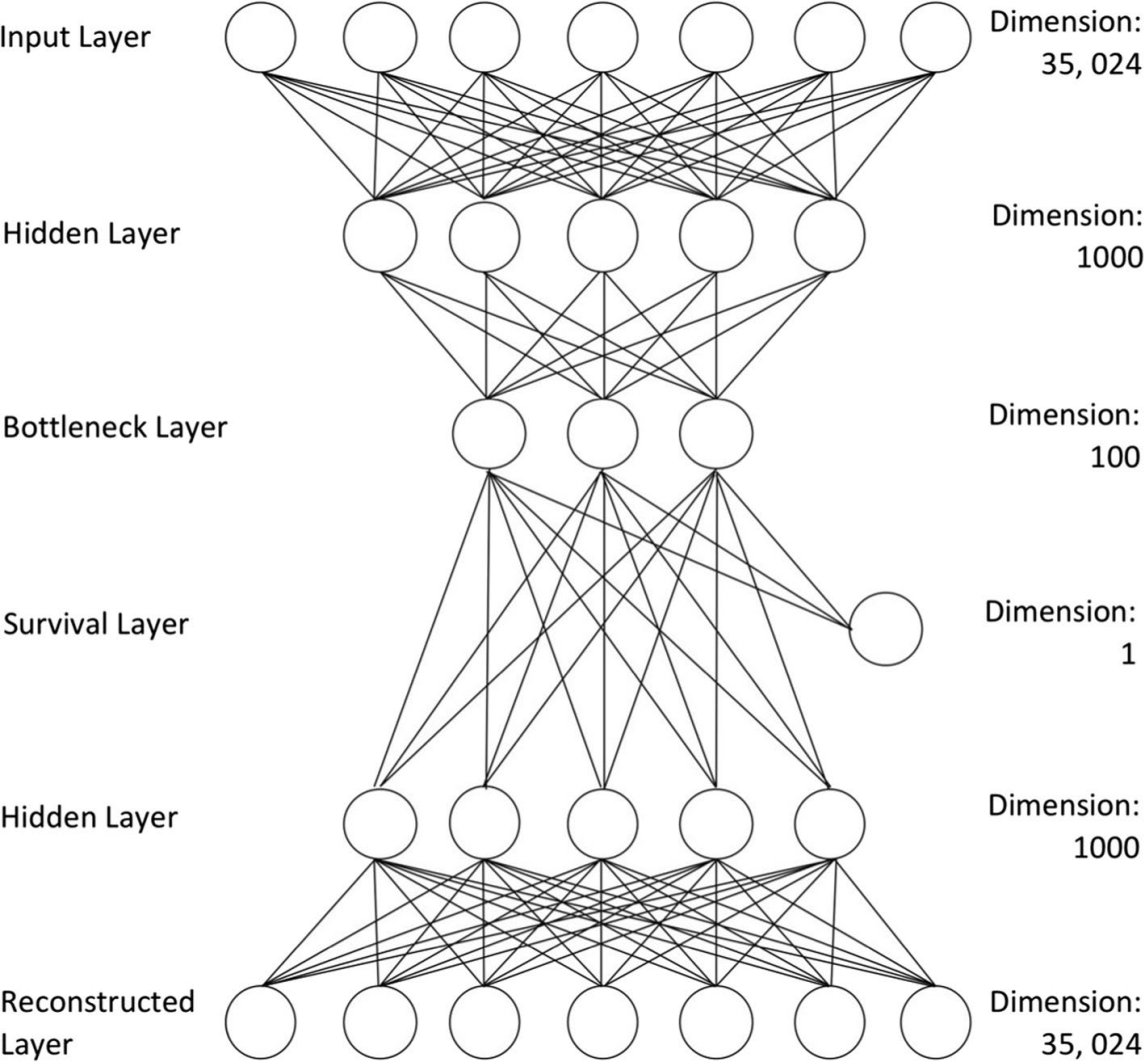
Custom autoencoder construction. The construction of the custom autoencoder with hidden layers, a bottleneck layer and a survival branch of dimension 1

Survival-based losses were explored to see if embedding survival analysis techniques directly into the autoencoder training can produce a latent space which, when clustered, produces better (in terms of survival) and more biologically meaningful groupings. Like Bello et al. [9], a branch of dimension 1 stemming from the bottleneck layer was added to the custom autoencoder (Fig. 1) and a Cox partial likelihood inspired loss L_S_ was applied. For losses using L_S_, survival information was used to sort the samples in descending order of survival before being fed to the autoencoder for the functioning of the survival loss implementation. For losses using L_S_, samples were not shuffled during training for the purposes of maintaining the survival ordering. The formula for calculating L_S_ is presented in Eq. 4_._

4 $$\begin{aligned} \log L\left( \beta \right) & = \mathop \sum \limits_{i = 1}^{n} \delta_{i} \left\{ {\beta^{\prime } z_{i} - \log \mathop \sum \limits_{{j \in R\left( {t_{i} } \right)}} e^{{\beta^{\prime}z_{j} }} } \right\} \\ L_{S} & = - \mathop \sum \limits_{i = 1}^{n} \delta_{i} \left\{ {W^{\prime } \phi \left( {x_{i} } \right) - \log \mathop \sum \limits_{{j \in R\left( {t_{i} } \right)}} e^{{W^{\prime } \phi \left( {x_{j} } \right)}} } \right\} \\ \end{aligned}$$

The $\log L\left( \beta \right)$ estimates the coefficients, denoted by $\beta$, of predictor variables represented by vector $z$. $\delta$ is an indication of whether subject $i$ is alive (0) or dead (1) and $R\left( {t_{i} } \right)$ is the risk set (subjects still alive at the time subject $i$ died). Coefficients describe the effect size of a particular variable, with positive $\beta$ suggesting a worse prognosis and negative $\beta$ suggesting a protective effect for that variable. For example, in a Cox model a positive coefficient for a variable such as age could mean that increasing age results in poorer prognosis. In L_S_, $W^{\prime } \phi \left( {x_{i} } \right)$ represents the single scaler output for the input of sample $i$ from the single dimension branch stemming from the bottleneck layer. Like before [9], L_S_ is combined with L_R_ forming: L_RS_ = αL_R_ + βL_S_. For both, L_RC_ and L_RS_ different parameter values were evaluated, with α = 0.25 and β = 0.75 found to be the optimal combination that allowed for the largest portion of the loss to be dedicated to the losses custom purpose.

**Fig. 2:**
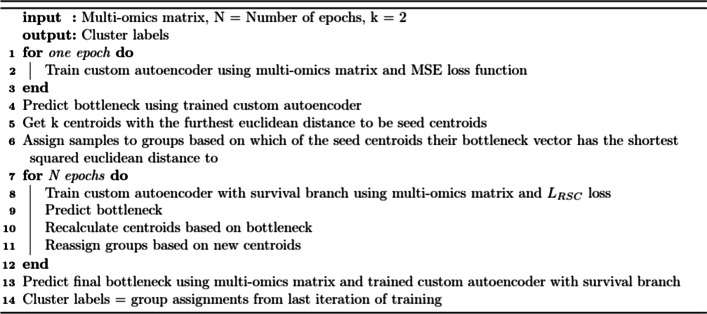
Pseudocode demonstrating the flow of training an autoencoder with L_RSC_ loss. The custom autoencoder is initially trained with an MSE loss in order to predict a bottleneck from which to identify seed centroids and group assignments which can then be used in the L_RSC_ loss during training. After each epoch training with L_RSC_ the centroids and group assignments are updated

Finally, it was investigated whether combining a cluster-based loss with a survival-based loss could produce a latent space which, when clustered, produced prognostic groups that were both significantly different in survival and biologically insightful. A combination cluster and survival loss was proposed: L_RSC_ = αL_R_ + βL_S_ + γL_C_ with the optimal parameters values set as α = 0.25, β = 0.50 and γ = 0.25. Pseudocode of the training using L_RSC_ is shown in Fig. 2. For all losses the entire dataset was used during the training and predicting phases. Loss stabilisation occurred at around 40 epochs for L_RC_, L_RS_ and L_RSC_ so this was considered to be optimal for these losses. After autoencoder training, the entire matrix was passed through the trained autoencoder's encoder function to produce a 352 × 100 matrix, a bottleneck vector for each sample.

It can be noted that L_R_ was included in each of the three loss functions. This is because L_R_ is used as a reconstruction loss in autoencoder models, which ensures that key information from the original data representation is encoded in the compressed bottleneck representation. Removing L_R_ from the loss would allow the bottleneck to diverge into a representation which is completely irrelevant to the original input data. That is why the final loss function needs to be balanced between L_R_ and L_C_/L_S_.

### Identifying prognostic subgroups and key features

Latent spaces produced by each model were clustered to identify subpopulations of patients with the aim that they would differ in survival (i.e. prognostic subgroups). For the baseline BCE and MSE losses, univariate Cox models were used, as before [6], to filter features for significance before clustering (unlike the other losses where all features were utilised during clustering). Thus, bottleneck feature selection was conducted after training. For the BCE and MSE models, the coxph function from the R survival library [18] was used to construct a univariate Cox model for each bottleneck feature as before [6]. Those features resulting in a significant model (log-rank test, *P*-value < 0.05) were selected and brought forward to the clustering phase. For those loss functions which utilized L_S_ or L_C_, all 100 bottleneck features were used to group patients as a part of the training process of the autoencoder. For BCE, MSE, and L_RS_ their cluster labels were created by clustering the relevant features using the KMeans function from Scikit-learn (16) (full algorithm with kmeans++ initialisation). For L_RC_ and L_RSC_ the group assignments were determined using the final centroids after the last training iteration. The quality of clusters was assessed using the Silhouette score, estimated using the silhouette_score function from Scikit-learn [16]. The Silhouette score can range from 1 to − 1 where 1 is the best score and indicates confidence in sample assignment to a cluster. The difference in survival between clusters was measured using a survival analysis with the pairwise_logrank_test function from lifelines [19]. The KaplanMeierFitter function from lifelines [19] was used to produce Kaplan Meier survival curves for the clusters.

**Fig. 3:**
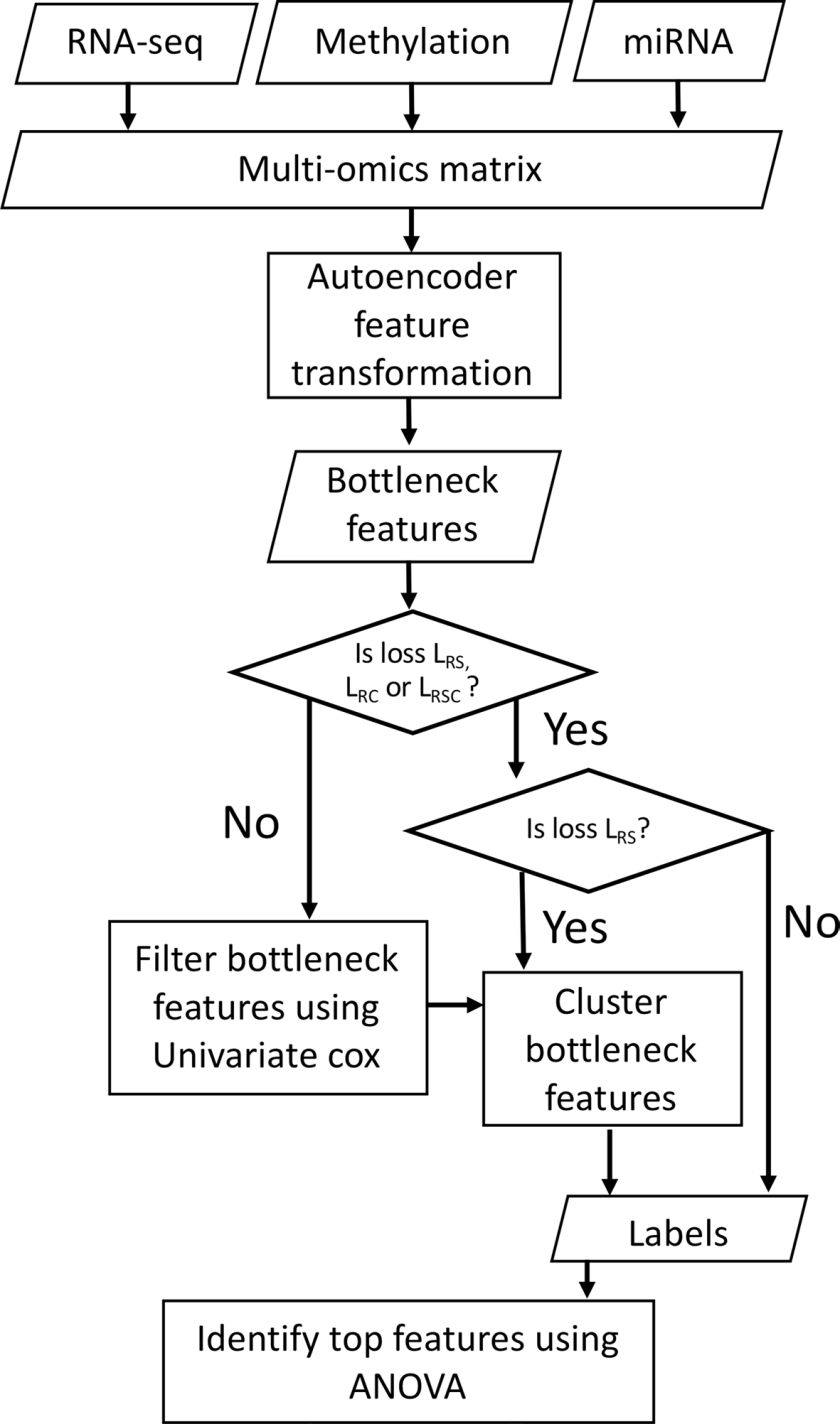
A flowchart of the autoencoder training process. The flowchart demonstrates how multi-omics data is combined into a single matrix, before being transformed by the autoencoder to produce bottleneck features. If the loss is BCE or MSE these bottleneck features are reduced by univariate Cox models as in the baseline before clustering to create group labels. For L_RC_ and L_RSC_ group labels from the final iteration of autoencoder training are used whereas for L_RS_ group labels are derived by clustering all bottleneck features. Using the identified cluster labels the top original omics features are identified for the run using ANOVA

Using cluster labels determined for each model in each run, the most important omics features in differentiating clusters were identified (Fig. 3). Before identifying the key features differentiating clusters, the three omics sets initially underwent a two-step scaling process, like that used in the baseline [6]. First, all omics types underwent median norm scaling. RNA-seq and methylation then underwent robust scaling using the RobustScaler from Scikit-learn [16], with miRNA undergoing unit norm scaling. An analysis of variance (ANOVA) was then performed with the f_oneway function from scipy [20]. Randomness introduced by factors such as the initialisation of weights could lead to slightly different cluster labels being identified and therefore different top features being selected each time the pipeline is run. For this reason, the entire pipeline was run 10 times for each model, with clusters being evaluated and top features derived. To derive top features from the ANOVA, the *P*-values were sorted from smallest to largest for each omics type. Of the top 10% of features, only those that had a significant *P*-value following correction for multiple testing of ten runs using Bonferroni (*P*-value < 0.005) were selected. For RNA-seq, 10% of features before filtering for significance was approximately 1561, for methylation this was approximately 1899 and for miRNA this was approximately 42. The top features identified by each of the ten runs of the different models were compared, and frequently identified features in six, eight and all ten runs quantified. While some features may have been identified by a small number of runs in a number of losses, the focus was on those features that were consistently identified. Thus, a feature was designated as robust if it was consistently identified i.e. it appeared as a top feature in all 10 runs for a loss. The robust omics features that were consistently identified as top features for each loss were compared to see if there were any features consistently detected by L_RSC_ that were not consistently identified by the other losses. The consistently identified omics features from each loss were also compared with the top omics features identified by the baseline paper’s ANOVA as these differed from the recreation in this work.

### Gene-enrichment and functional annotation analysis and prognostic validation

A Gene-enrichment and Functional Annotation Analysis (GEFA) was conducted using only those mRNA features consistently identified by L_RSC_. GEFA was implemented with DAVID, the online bioinformatics portal [21]. Entrez Ids were converted to gene symbols using DAVID and a KEGG pathway analysis was performed using significant mRNA features that were successfully mapped. An EASE score threshold of 0.10 was used for significance. In each omics type, the consistent top features identified by L_RSC_ were then used in a survival analysis to assess their prognostic significance. Using the scaled data, median expression was used for patient separation in the Kaplan meier survival curve. The prognostic significance of resulting clusters was assessed using the log-rank test (*P*-value < 0.05). The low expression cluster included those whose values were below or equal to the median and the high expression cluster included those whose values were above the median.

Using the pre-processed omics data before scaling, significant mRNA and methylation features consistently identified by L_RSC_ were visualised in prognostic clusters using heatmaps. Heatmaps were produced using Seaborn [22] with expression shown as a z-score, which is a representation of standard deviations of expression of each gene from the mean. Scale was limited to − 3 + 3 using vmin and vmax in the clustermap function in Seaborn to improve the visualisation of differences between the prognostic clusters being presented.

## Results

### Cluster evaluation

**Fig. 4:**
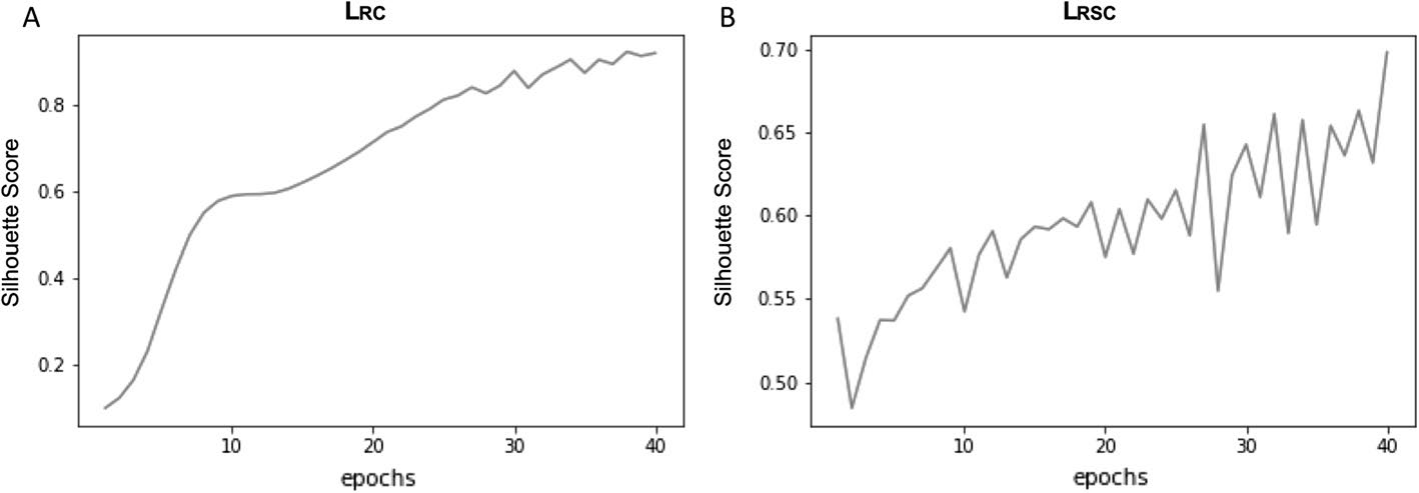
Silhouette scores per epoch for best run (by Silhouette score) of L_RC_ and L_RSC_. A comparison of the Silhouette scores for each epoch for the best run (as determined by Silhouette score) for L_RC_ (0.92) and L_RSC_ (0.7)_._ **A** For L_RC_ the Silhouette score rises in a steep and linear fashion due to the heavy influence of the clustering loss L_C_. **B** L_RSC_ shows an upward trend, albeit with more fluctuations due to the heavy weight of the survival-based L_S_ loss compared to the clustering-based L_C_

Cluster quality metrics were compared between models (Table 2). The clusters obtained by the baseline models BCE and MSE had lower Silhouette scores (0.18–0.31), indicating poorer cluster quality, i.e. more heterogeneous. For BCE and MSE, the number of significant bottleneck features to be used in clustering following filtering, as identified by the univariate Cox models, was quite low and ranged from 11 to 21 out of the bottleneck dimension of 100. By comparison, some of the highest Silhouette scores were achieved by L_RC_, demonstrating that the k-means inspired loss produced better quality clusters compared to the other losses. This provided greater confidence in sample assignment to clusters by the L_RC_ and indicated that they comprised of more homogeneous and consequently more biologically meaningful groupings. For loss functions using L_C_, the bottleneck layer was clustered according to group labels derived after each epoch. For L_RC_ and L_RSC,_ the Silhouette score of the bottleneck improves as epochs increase (Fig. 4). With the heavy influence of L_C_ the ascent of the Silhouette score in L_RC_ (Fig. 4A) is smoother than L_RSC_ (Fig. 4B). The Silhouette score steadily rises with continued training with L_RC_, thereby indicating a continuous improvement in sample assignment for both models.

**Table 2:** A comparison of the cluster quality metrics for each loss, including the highest and lowest Silhouette scores and the highest and lowest log-rank *P*-values across ten runs for each loss function

Method	Loss Function	Log-rank *P*-value (lowest)	Log-rank *P*-value (highest)	Silhouette score (highest)	Silhouette score (lowest)
Baseline-binary cross entropy loss	BCE	6.68E−04	1.38E−01	0.29	0.2
Baseline-mean squared error loss	MSE/L_R_	4.07E−04	1.51E−01	0.31	0.18
Clustering loss	L_RC_	9.11E−02	3.83E−01	0.92	0.59
Survival loss	L_RS_	1.89E−96	6.70E−62	0.77	0.62
Combined survival and Clustering loss-hybrid model	L_RSC_	1.55E−77	2.62E−61	0.7	0.59

A summary of the highest and lowest Silhouette scores and log-rank *P*-values across 10 runs for each loss function. The log-rank *P*-values for MSE and BCE varied between significant and non-significant, whereas the survival-based losses produced the lowest log-rank *P*-values. Silhouette scores for MSE and BCE remained below 0.4, indicating low confidence in group assignments whereas the clustering-based loss L_RC_ was able to produce the highest Silhouette scores, indicating high confidence in group assignments

**Fig. 5:**
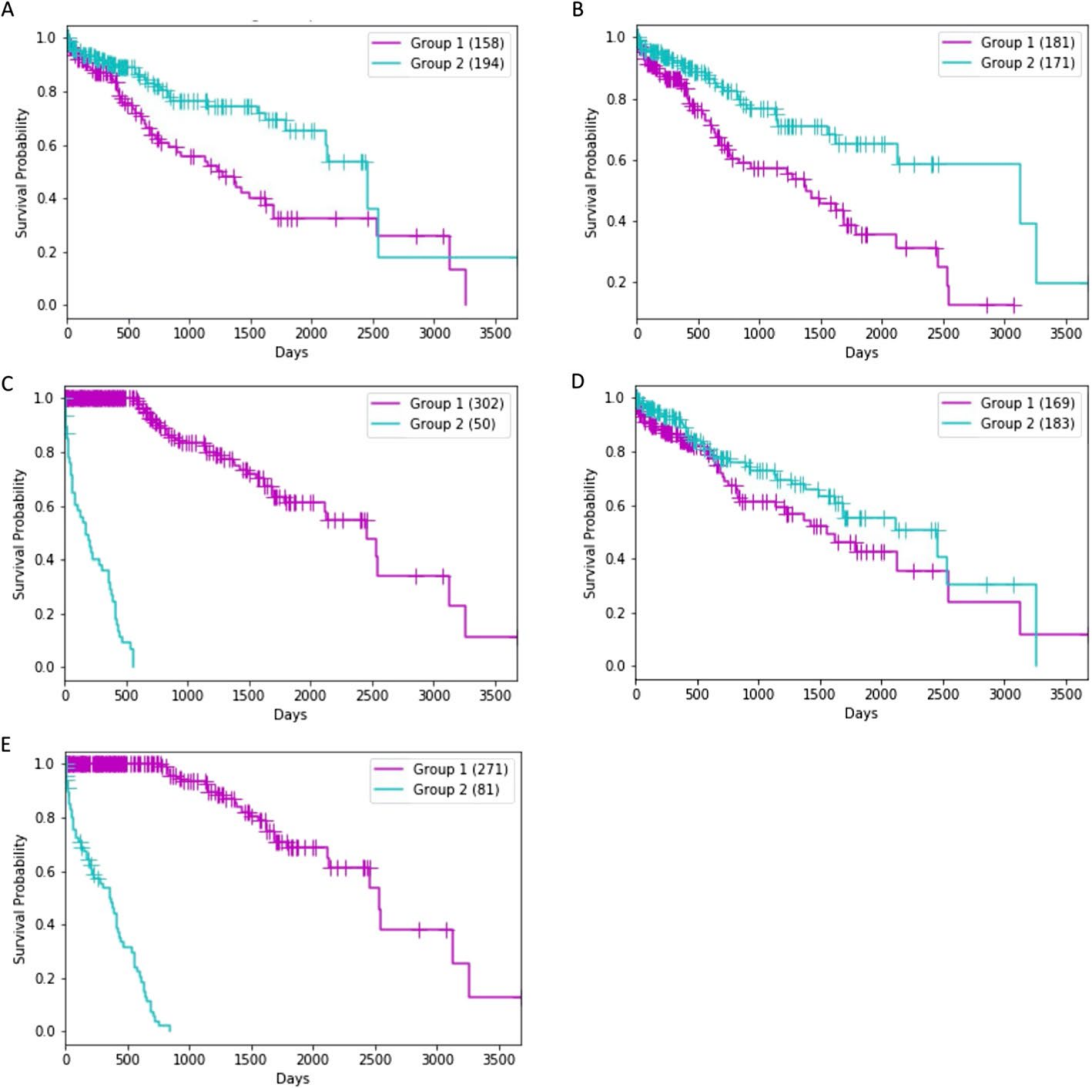
Comparing prognostic significance of groups identified by clustering relevant deep learning features produced by each loss function. **A** BCE (*P* = 6.68E-04); **B** MSE (*P* = 4.07E-04); **C** L_RS_ (*P* = 1.89E-96); D) L_RC_ was non-significant (*P* = 9.11E-02) and E) L_RSC_ (*P* = 1.55E-77). Kaplan–Meier plot results presented are based on the best run out of ten as determined by the log-rank *P*-value

Clusters were also assessed for their significance in terms of prognosis (Table 2). Clusters identified by the best run using the log-rank test *P*-value for each loss function were examined (Fig. 5). Separation between prognostic survival groups was greatest for L_RS_ (1.89E−96; Fig. 5C) followed by L_RSC_ (Fig. 5E; 1.55E−77). Size split between clusters differed between models. BCE, MSE and L_RC_ all had a relatively even split in group sizes, which were 158 v 194, 181 v 171 and 169 v 183, respectively (Figs. 5A, B, D). The survival-based loss produced a more uneven split (302 v 50) compared to that created by the L_RSC_ loss (271 v 81). In general, those losses which included L_S_ had a more uneven split in group sizes compared to the other losses. Cluster membership for all models did not have any evidence that they related to HCC disease grade or risk factors (Table 3).

**Table 3:** Clinical characteristics and risk factors of the TCGA cluster members for the different loss functions

		BCE		MSE		L_RS_		L_RC_		L_RSC_	
		C1 (158)	C2 (194)	C1 (181)	C2 (171)	C1 (302)	C2 (50)	C1 (169)	C2 (183)	C1 (271)	C2 (81)
Risk factors	Alcohol consumption	59	55	69	45	103	11	64	50	89	25
	Hepatitis B	45	58	46	57	88	15	54	49	86	17
	Hepatitis C	24	29	30	23	44	9	29	24	40	13
	Non-alcoholic fatty liver disease	5	14	9	10	18	1	11	8	18	1
	Hemochromatosis	2	3	3	2	4	1	2	3	3	2
	Alpha-1 Antitrypsin Deficiency	0	1	0	1	0	1	1	0	0	1
	No history of risk factors	36	47	39	44	68	15	36	47	59	24
	Other	11	10	14	7	16	5	9	12	11	10
Grade	Grade I	14	38	29	23	49	3	27	25	44	8
	Grade II	70	95	85	80	142	23	85	80	129	36
	Grade III	65	55	62	58	100	20	50	70	87	33
	Grade IV	8	3	4	7	7	4	5	6	7	4
	Not available	1	3	1	3	4	0	2	2	4	0

Clinical characteristics and risk factors of the TCGA cluster members for the BCE, MSE, L_RC_, L_RS_ and L_RSC_ loss functions. Results are taken from the best run out of ten for each loss (as determined by log-rank *P*-value)

Univariate Cox models created using bottleneck features from the best run of L_RSC_ by log-rank test *P*-value are presented in Table S1. A hazard ratio of one suggests that there was no difference in survival between groups. Hazard ratios for all of the 100 bottleneck features produced by the combined loss L_RSC_ differed greatly from one (range: 2.29E−45 to 2.82E+45; Additional file 1: Table S1). This provided evidence that all the L_RSC_ bottleneck features were highly explanatory for HCC survival and conveyed either a massively increased risk, or a massively reduced risk. This indicated that by using the combined loss we can learn a new joint representation of the multi-omics data that is highly informative for the task of prognostic subgroups identification. Using the new data representation (i.e. bottleneck features), we were able to identify new subgroups with greater differences in survival (log-rank *P*-value) and more biologically meaningful (Silhouette score) in comparison to the baseline.

### Top features identified by the loss functions

Top features frequently identified in the three omics types for 6, 8 and 10 runs were examined for each loss function (Table 4). The baseline BCE model achieved the greatest number of consistent mRNA features across 10 runs. For miRNA and methylation, the clustering loss L_RC_ produced by far the greatest number of overlapping top features across 10 runs. This demonstrates that L_RC_ is robust to randomness when it comes to identifying these types of omics features across runs. In total, 377 mRNA features were consistently identified by the hybrid model L_RSC_*.* Of these, 231 (61.27%) were novel i.e. they were only consistently identified by L_RSC_ compared to the features consistently identified by the other losses as well as the baseline paper’s top 100 RNA-seq features derived from ANOVA. The results of the GEFA with the 229 of the 231 genes that mapped in DAVID identified seven pathways including cell cycle and DNA replication (Table 5).

**Table 4:** Common features identified across runs for the different omics data types

Omics type	No. of runs	Loss functions				
		BCE	MSE	L_RC_	L_RS_	L_RSC_
mRNA	6 runs	1296	1319	1268	422	888
	8 runs	929	986	742	256	633
	10 runs	451	295	439	124	377
miRNA	6 runs	31	34	36	9	16
	8 runs	22	21	20	1	9
	10 runs	12	1	15	1	3
Methylation	6 runs	1676	1593	1794	647	1135
	8 runs	1252	1203	1388	330	856
	10 runs	675	520	1097	89	434

A summary of the common features identified for the different omics data types (mRNA, miRNA, methylation) across six, eight and ten replicate runs of models with the five different losses

**Table 5:** Results of the Gene-enrichment and Functional Annotation Analysis for mRNAs of interest identified by L_RSC_

KEGG pathway	Count	%	*P*-value/EASE	Genes
Aminoacyl-tRNA biosynthesis	7	3.06	1.40E−04	*YARS, LARS, PARS2, MARS, TARS, HARS, QRSL1*
Cell cycle	8	3.49	7.49E−04	*ORC1, PLK1, CUL1, TTK, MCM6, SMC1A, BUB1, MAD2L1*
Purine metabolism	7	3.06	2.02E−02	*POLA1, ADSL, RRM2, PRIM1, PPAT, PDE2A, GMPS*
Oocyte meiosis	5	2.18	4.63E−02	*PLK1, CUL1, SMC1A, BUB1, MAD2L1*
RNA transport	6	2.62	5.86E−02	*NDC1, NUP155, GEMIN5, GEMIN8, EIF2S1, NUP37*
Alanine, aspartate and glutamate metabolism	3	1.31	6.76E−02	*ADSL, PPAT, CAD*
DNA replication	3	1.31	7.11E−02	*POLA1, PRIM1, MCM6*

A summary of the results of the Gene-enrichment and Functional Annotation Analysis (GEFA) for the mRNAs of interest identified by L_RSC_. In all, 231 overlapping genes were only consistently identified by L_RSC_, 229 of these genes mapped in DAVID and were included in the GEFA

**Fig. 6:**
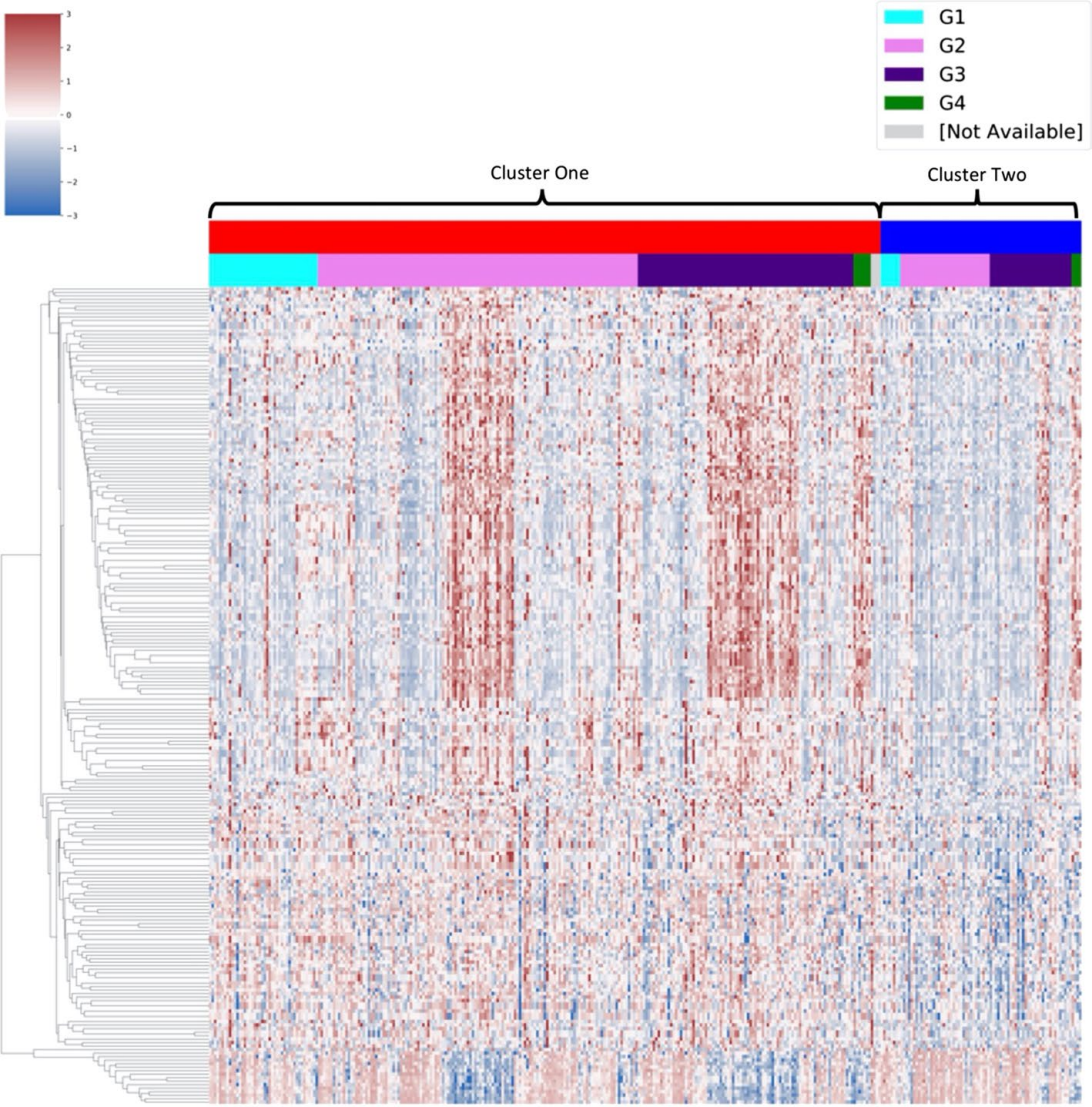
Visualisation of the methylation patterns of robust significant methylation features that were consistently identified by L_RSC_. A visualisation of the methylation patterns of the 233 significant methylation features consistently identified across all ten runs by L_RSC_. Cluster labels were taken from the best run of L_RSC_ as determined by log-rank P-value. Methylation is shown as a z-score. Scale was limited to − 3 + 3 using vmin and vmax in the clustermap function in Seaborn to improve visualisation of differences between prognostic clusters. The distribution of HCC grades between clusters appears to be relatively even

Fifteen miRNAs were identified as robust features. Two miRNAs, hsa-let-7g and hsa-mir-550a-1, were also novel, i.e., they were consistently identified by L_RSC_ but not consistently identified by the other losses or by the baseline paper’s top 50 miRNA as identified by ANOVA. These miRNAs were not prognostic, however, as their expression did not show a significant difference in separation of patients in terms of survival. For methylation, 328 features were consistently identified by L_RSC_ compared to the other losses or the paper’s top methylation features as identified by ANOVA. Of these 233 were prognostic as the log-rank test was significant using a median expression for survival group separation (Additional file 1: Table S2). For these 233 features, a subtle difference in the expression patterns between clusters was observed (Fig. 6).

**Fig. 7:**
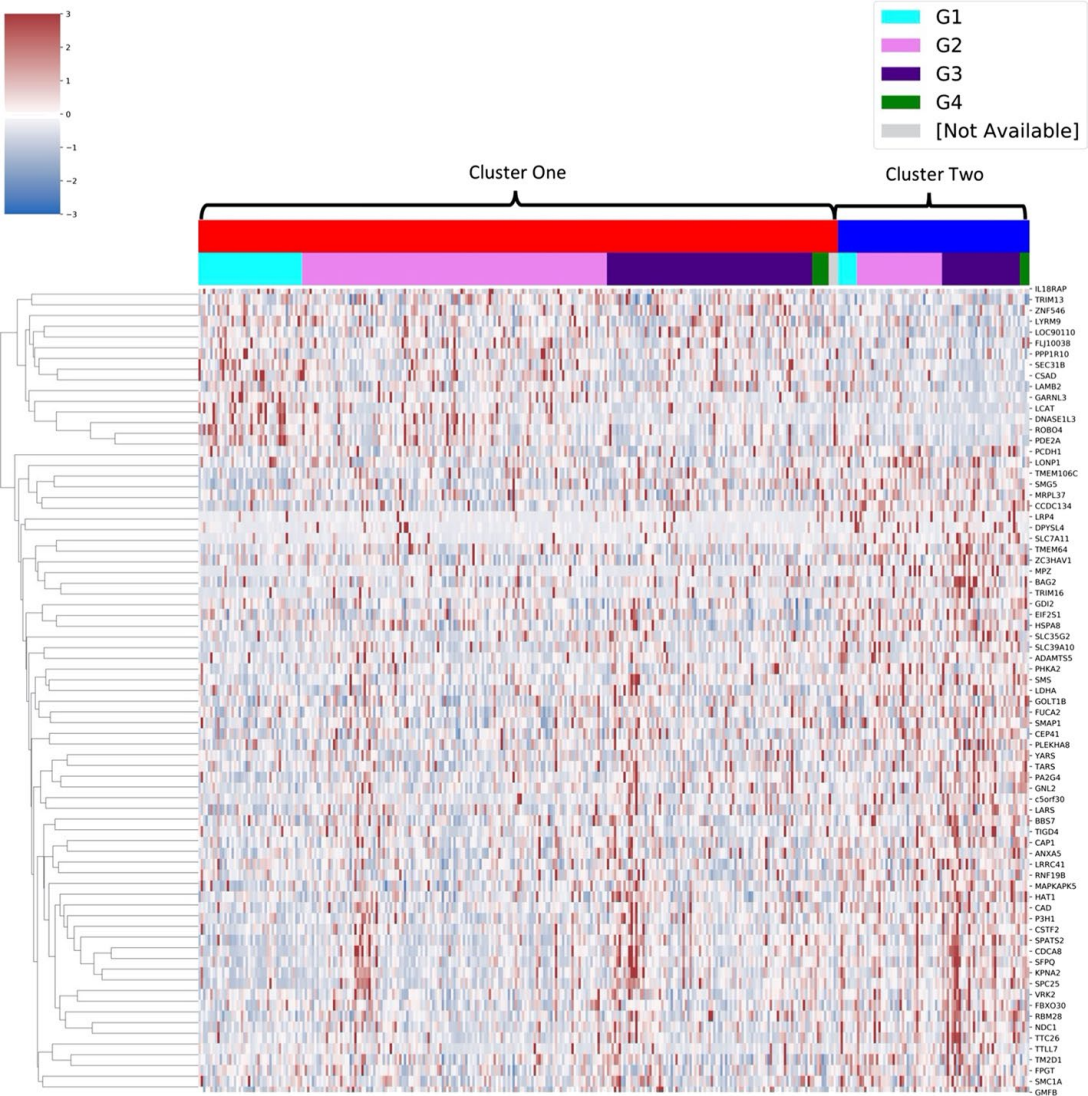
Visualisation of the 75 mRNAs consistently identified by L_RSC_ that were significant for prognosis. A visualisation of the gene expression patterns of the 75 mRNAs that were consistently identified across all ten runs by L_RSC_ and were significant for prognosis using a median split in gene expression. Cluster labels were taken from the best run of L_RSC_ as determined by log-rank *P*-value. Expression is shown as a z-score. Scale was limited to − 3 + 3 using vmin and vmax in the clustermap function in Seaborn to improve visualisation of differences between prognostic clusters. The distribution of HCC grades between clusters appears to be relatively even

Of the 231 mRNAs of interest, 75 significantly grouped patients in terms of survival in the TCGA HCC cohort when separated by median expression (Table 6). Expression of these 75 mRNAs varied subtly between prognostic clusters (Fig. 7). The 75 mRNAs were validated using a survival analysis carried out online using the HCCDB interactive tool. In total, 29 of the 75 mRNAs were also significant for prognosis in the LIRI-JP HCC cohort (Table 6).

**Table 6:** Results of the survival analysis of the 75 significant mRNAs identified by L_RSC_

Entrez ID	Symbol	TCGA median log-rank	LIRI-JP median log-rank
**5036**	***PA2G4***	1.47E−03	5.30E−06
**3838**	***KPNA2***	1.40E−02	1.30E−05
**57405**	***SPC25***	3.05E−02	1.55E−05
**8550**	***MAPKAPK5***	2.79E−02	4.44E−05
**55143**	***CDCA8***	2.61E−02	7.28E−05
**1776**	***DNASE1L3***	2.16E−02	2.49E−04
**54538**	***ROBO4***	1.37E−02	2.98E−04
**5138**	***PDE2A***	6.89E−03	3.17E−04
**25956**	***SEC31B***	1.85E−03	3.22E−04
**6421**	***SFPQ***	8.09E−04	1.37E−03
**55706**	***NDC1***	4.12E−02	2.01E−03
**51380**	***CSAD***	1.70E−02	2.02E−03
**2665**	***GDI2***	4.79E−02	2.78E−03
**8520**	***HAT1***	1.35E−02	3.04E−03
**2519**	***FUCA2***	4.19E−02	3.06E−03
**51026**	***GOLT1B***	2.92E−03	6.00E−03
**29889**	***GNL2***	2.63E−03	7.94E−03
**790**	***CAD***	2.63E−02	1.03E−02
**8243**	***SMC1A***	2.27E−02	1.07E−02
**339327**	***ZNF546***	2.67E−02	1.14E−02
**1478**	***CSTF2***	6.14E−03	1.50E−02
**79022**	***TMEM106C***	4.48E−04	1.64E−02
**84253**	***GARNL3***	2.01E−02	2.14E−02
**9361**	***LONP1***	2.18E−02	2.27E−02
**23381**	***SMG5***	2.95E−02	2.67E−02
**9532**	***BAG2***	1.32E−02	2.74E−02
**55131**	***RBM28***	1.23E−02	2.91E−02
**4038**	***LRP4***	1.06E−03	4.07E−02
**83941**	***TM2D1***	3.81E−02	4.84E−02
90355	*c5orf30*	8.13E−03	6.17E−02
23657	*SLC7A11*	2.26E−03	7.32E−02
10489	*LRRC41*	4.73E−02	8.07E−02
65244	*SPATS2*	3.16E−02	8.16E−02
3913	*LAMB2*	8.27E−03	8.52E−02
7444	*VRK2*	1.95E−02	8.66E−02
8807	*IL18RAP*	1.34E−03	8.75E−02
79739	*TTLL7*	3.94E−02	8.88E−02
2764	*GMFB*	7.68E−03	1.00E−01
51253	*MRPL37*	1.81E−02	1.02E−01
1965	*EIF2S1*	5.40E−03	1.22E−01
127544	*RNF19B*	6.66E−03	1.32E−01
60682	*SMAP1*	4.86E−03	1.46E−01
3931	*LCAT*	1.92E−02	1.48E−01
56829	*ZC3HAV1*	4.36E−02	1.70E−01
5514	*PPP1R10*	4.66E−02	1.81E−01
8565	*YARS*	5.54E−04	1.90E−01
55056	*FLJ10038*	3.73E−02	2.03E−01
10626	*TRIM16*	2.97E−02	2.04E−01
10487	*CAP1*	4.08E−02	2.29E−01
10570	*DPYSL4*	1.36E−02	2.35E−01
201229	*LYRM9*	5.58E−03	2.66E−01
4359	*MPZ*	3.41E−02	3.00E−01
79989	*TTC26*	8.51E−04	3.53E−01
308	*ANXA5*	1.85E−04	4.24E−01
57181	*SLC39A10*	2.64E−02	4.35E−01
5256	*PHKA2*	4.46E−02	4.40E−01
6611	*SMS*	1.48E−02	4.49E−01
95681	*CEP41*	1.59E−02	4.70E−01
64175	*P3H1*	1.38E−03	4.81E−01
6897	*TARS*	2.13E−02	5.20E−01
10206	*TRIM13*	1.47E−02	5.23E−01
55212	*BBS7*	9.59E−03	5.35E−01
3939	*LDHA*	3.08E−04	5.37E−01
201798	*TIGD4*	3.85E−02	5.43E−01
169200	*TMEM64*	8.06E−03	5.66E−01
84725	*PLEKHA8*	3.97E−02	5.96E−01
5097	*PCDH1*	1.37E−02	6.23E−01
84085	*FBXO30*	6.83E−03	6.56E−01
8790	*FPGT*	2.03E−02	7.22E−01
79879	*CCDC134*	2.67E−04	8.10E−01
3312	*HSPA8*	3.49E−03	9.06E−01
11096	*ADAMTS5*	4.80E−04	9.14E−01
51520	*LARS*	2.06E−03	9.46E−01
80723	*SLC35G2*	3.38E−02	9.46E−01
90110	*LOC90110*	4.47E−02	NA

A summary of the results of the survival analysis of the mRNAs identified by L_RSC_. A total of 75 genes were prognostic in the TCGA HCC cohort as indicated by a significant log-rank result; 29 of these were also prognostic in the LIRI-JP cohort, indicated here in bold. NA—Not applicable for testing as gene not available in validation dataset

## Discussion

In future, greater data volumes generated by testing for a wider array of markers will be routinely available for all patients and used to assist with their clinical diagnosis and stratification into groups based on disease subtype. Implementing a precision medicine approach, patients may then receive tailored treatments thereby improving their overall survival. Developing analytical methods for prognostic group identification is therefore vital to further understand key drivers in cancers of unmet need. This study explored a novel deep learning approach for prognostic group identification. For the first time, both cluster quality and survival metrics were included into a combined loss function for training an autoencoder model. The hybrid model, L_RSC_ had bottleneck feature representation that was tailored specifically for grouping HCC patients by prognosis. All one hundred bottleneck features identified by L_RSC_ were significant for survival, indicating their strong predictive power for increased / reduced risks to HCC survival. By comparison, only 11–21 significant bottleneck features could be identified by the baseline models following training. The L_RSC_ latent space, when clustered, provided more insightful patient groups than the baseline method [6]. Pipelines utilising survival information, either to filter bottleneck features after network training (BCE, MSE) or directly within the loss function (L_RS_, L_RSC_) were able to separate patients in terms of survival more significantly than the unsupervised L_RC_ loss, as assessed using the log-rank test. As expected, the clustering loss L_RC_ produced quality clusters that had higher Silhouette scores (best = 0.92) than the other loss functions. This was likely due to the high weight of L_C_ in the loss, meaning that the latent spaces produced were more complementary to clustering. It was interesting to note that applying L_RS_ led to very distinct clusters in terms of survival but it also gave good quality clusters in terms of the Silhouette score (best = 0.77). However, the structure of the clusters obtained by L_RC_ and L_RS_ differed. The combined loss L_RSC_ produced a set of clusters that balanced the requirements of both L_C_ and L_S_. In this study, the Silhouette score was lower for L_RSC_ (0.59–0.7) than for L_RS_ (0.62–0.77). While L_RSC_ wasn’t able to produce lower *P*-values in the log-rank test and higher Silhouette scores than L_RS,_ combining L_C_ with L_RS_ produced more robust and biologically meaningful clusters. Baseline models BCE and MSE had low Silhouette scores (0.18–0.31) indicating less accurate sample assignment to clusters.

Despite the complexity of the patient cohorts and their underlying conditions in addition to HCC, the hybrid model successfully identified robust features of biological and prognostic significance in different omics data types (mRNA, miRNA, methylation). Features were identified consistently across ten runs of the model. This included 377 mRNAS, 231 of which were novel compared to the other losses explored and those listed in the baseline paper’s top 100 mRNA features as identified by ANOVA. A total of 75 of the 231 mRNAs were significant for prognosis in the TCGA cohort when groups were separated by median expression. Amongst the mRNAs identified by L_RSC_ was LCAT. Low expression of LCAT has been linked to poor survival in HCC, and furthermore it has been used in prognostic models for the disease [23, 24]. In all, 29 of the 75 (38.67%) mRNA features significant for prognosis in the TCGA cohort were also significant in the LIRI-JP cohort. The complexity of underlying risk factors of the HCC patients in the different cohorts may have accounted for why only a proportion of features could be validated as prognostic in both cohorts. A total of 15 robust miRNAs were also identified by the hybrid model. Of the two miRNA features of interest that were novel, hsa-let-7g has been linked to inhibition of HCC cells proliferation [25]. The other novel miRNA identified, hsa-mir-550a-1, has not previously been linked with HCC and would warrant experimental investigation perhaps. Of the 328 methylation features consistently identified by L_RSC,_ 233 were significant for prognosis. Some of these genes have been previously linked with HCC, including RICTOR, which was found to be dysregulated in cancers, including HCC [26].

It was interesting to note the differences in the expression / methylation profiles of the top omics features between the prognostic clusters derived by the hybrid loss. These features tended to exhibit only low and subtle expression and methylation differences between prognostic groups. Thus, it remains to be established whether the features identified by the hybrid approach could have utility in diagnostics as biomarkers with current approaches using expression or pyrosequencing assays. Also a limitation of the method is that the methylation specific CpG site information was collapsed during data pre-processing, therefore changes to this stage would be required to identify key methylation features that differed in order to be able to design a diagnostic assay.

Nevertheless, mRNA and methylation patterns were differential between prognostic groups, which would warrant further testing for their clinical application as targeted therapies for HCC or other. It may be that other biological processes might distinguish how these genes function differently between prognostic groups. For example, patient groups may differ in their epigenetic profiles or post-transcriptional processing or modification of these mRNAs, whereby some are silenced whilst others go on to become a protein with functional impact. Thus, in future, assays that focus on other RNA processes related to those features perhaps may prove more useful for diagnostics compared to traditional tests.

Certainly the hybrid approach developed here identified a suite of unbiased features that may be more representative of the aetiology. This is because only the significant features in the top 10% of the omics ANOVA results for each run were considered here. Also examining the key features consistently identified across ten multiple runs should have prevented any oversight of the most important features of interest. In this work, a single Gene-enrichment and Functional Annotation Analysis was conducted on the features that were most different between clusters. The reason for this was to focus on uncovering the biological pathways that differed between prognostic patient groups. Prognostic subgroups for the losses did not appear to be explained by clinical characteristics such as disease grade or risk factors. Instead many of the robust features identified by the hybrid model that distinguished prognostic groups were novel and had not been previously described for HCC by similar models. Thus, further investigation of the robust features that distinguished prognostic groups could determine whether any of these genomic alterations that distinguished patients in groups would be of interest from a clinical perspective for diagnosing new HCC survival subtypes.

## Conclusions

Autoencoders trained using L_RS_ and L_RSC_ produced more statistically robust results. This work demonstrates that utilising a joint clustering and survival objective function can identify new patient subgroups that are prognostic and provide biological insights for target identification for therapeutics. This information is important for discovery within precision medicine and the development of new therapies for patient interventions. Future directions of this work would be the application of the proposed analysis pipeline to other cancers of poor clinical outcome, such as brain tumours (gliomas), or other diseases where survival and omics information is becoming more routinely available.

## Supplementary Information

**Additional file 1:** Supplementary Tables. **Table S1.** Results from the univariate cox models for each bottleneck feature for best run of LRSC. **Table S2.** Methylation features consistently identified in ten runs of LRSC that are significant for prognosis.

## Data Availability

TCGA data was downloaded using TCGA-Assembler 2 which can be accessed on https://github.com/compgenome365/TCGA-Assembler-2. LIRI-JP data was accessed using HCCDB which can be found on http://lifeome.net/database/hccdb/home.html. All source code developed by this study is publicly available at: https://github.com/aowens-code/DeepLearningSurvivalClustering.

## Abbreviations

HCC: Hepatocellular carcinoma
TCGA: The Cancer Genome Atlas
ICGC: International Cancer Genome Consortium
GEFA: Gene-enrichment and Functional Annotation Analysis
LIRI-JP: The Liver Cancer, Riken Japan
BCE: Binary cross-entropy
MSE: Mean squared error
L_C_: Clustering-based loss
L_S_: Survival based loss
L_R_: Reconstruction based loss
L_RC_: Clustering and reconstruction based combined loss
L_RS_: Survival and reconstruction_- _based combined loss
L_RSC_: Survival, clustering and reconstruction-based combined loss; hybrid model
