# Supplementary material for: Novel deep learning-based solution for identification of prognostic subgroups in liver cancer (Hepatocellular carcinoma)
Source: BMC Bioinformatics. 2021 Nov 24;22:563. doi: 10.1186/s12859-021-04454-4 (PMC8611905; doi:10.1186/s12859-021-04454-4)
Supplement: Supplementary file 1 — Additional file 1: Supplementary Tables. Table S1. Results from the univariate cox models for each bottleneck feature for best run of LRSC. Table S2. Methylation features consistently identified in ten runs of LRSC that are significant for prognosis. [file 12859_2021_4454_MOESM1_ESM.docx]

**Supplementary Tables**

**Table S1. Results from the univariate cox models for each bottleneck feature for best run of L_RSC_.**

| Bottleneck Feature | HR | (95% CI) | z | Pr(>\|z\|) |
| --- | --- | --- | --- | --- |
| 1 | 8.90E-35 | (9e-42 - 8.8e-28) | -9.54 | 1.42E-21 |
| 2 | 2.90E-34 | (1.71e-41 - 4.93e-27) | -9.09 | 9.77E-20 |
| 3 | 1.51E+34 | (5.64e+26 - 4.02e+41) | 9.02 | 1.88E-19 |
| 4 | 8.27E-28 | (3.26e-33 - 2.09e-22) | -9.82 | 8.91E-23 |
| 5 | 6.46E-32 | (3.46e-38 - 1.21e-25) | -9.75 | 1.91E-22 |
| 6 | 6.82E+31 | (1.91e+25 - 2.44e+38) | 9.52 | 1.70E-21 |
| 7 | 1.78E-17 | (2.73e-20 - 1.16e-14) | -11.7 | 2.00E-31 |
| 8 | 5.69E-35 | (4.36e-42 - 7.43e-28) | -9.43 | 4.00E-21 |
| 9 | 3.52E+37 | (4.3e+29 - 2.89e+45) | 9.3 | 1.42E-20 |
| 10 | 7.32E+29 | (7.57e+23 - 7.07e+35) | 9.78 | 1.38E-22 |
| 11 | 2.14E-33 | (6.3e-40 - 7.27e-27) | -9.8 | 1.08E-22 |
| 12 | 8.07E-33 | (2.13e-39 - 3.05e-26) | -9.56 | 1.15E-21 |
| 13 | 1.66E-34 | (1.26e-41 - 2.21e-27) | -9.3 | 1.47E-20 |
| 14 | 6.46E-32 | (2.96e-38 - 1.41e-25) | -9.64 | 5.20E-22 |
| 15 | 9.45E-32 | (5.37e-38 - 1.66e-25) | -9.74 | 2.13E-22 |
| 16 | 2.60E+33 | (3.91e+26 - 1.73e+40) | 9.6 | 8.07E-22 |
| 17 | 2.98E+26 | (5.16e+21 - 1.73e+31) | 10.9 | 1.21E-27 |
| 18 | 2.94E-36 | (1.17e-43 - 7.36e-29) | -9.41 | 4.85E-21 |
| 19 | 8.92E+32 | (2.46e+26 - 3.23e+39) | 9.85 | 7.14E-23 |
| 20 | 1.86E-32 | (6.04e-39 - 5.75e-26) | -9.58 | 9.38E-22 |
| 21 | 1.52E+22 | (8.2e+17 - 2.82e+26) | 10.2 | 2.29E-24 |
| 22 | 7.64E-37 | (1.94e-44 - 3.01e-29) | -9.32 | 1.17E-20 |
| 23 | 5.92E-33 | (1.21e-39 - 2.9e-26) | -9.44 | 3.67E-21 |
| 24 | 7.77E+26 | (5.73e+21 - 1.05e+32) | 10.3 | 9.71E-25 |
| 25 | 1.37E+42 | (6.68e+32 - 2.8e+51) | 8.87 | 7.34E-19 |
| 26 | 1.72E-20 | (3.08e-24 - 9.63e-17) | -10.3 | 4.85E-25 |
| 27 | 1.83E+31 | (1.08e+25 - 3.13e+37) | 9.83 | 8.15E-23 |
| 28 | 2.05E-24 | (8.14e-29 - 5.19e-20) | -10.5 | 5.32E-26 |
| 29 | 5.45E+26 | (5.39e+21 - 5.51e+31) | 10.5 | 1.18E-25 |
| 30 | 2.96E+31 | (1.16e+25 - 7.6e+37) | 9.62 | 6.28E-22 |
| 31 | 6.83E-24 | (2.26e-28 - 2.06e-19) | -10.1 | 3.86E-24 |
| 32 | 8.70E+39 | (3.59e+31 - 2.11e+48) | 9.34 | 1.00E-20 |
| 33 | 1.73E+18 | (8.6e+14 - 3.46e+21) | 10.8 | 2.67E-27 |
| 34 | 3.70E-32 | (2.46e-38 - 5.56e-26) | -9.97 | 1.98E-23 |
| 35 | 1.50E-21 | (1.47e-25 - 1.53e-17) | -10.2 | 2.40E-24 |
| 36 | 3.78E-29 | (1.99e-34 - 7.2e-24) | -10.6 | 5.01E-26 |
| 37 | 2.80E-18 | (2.8e-21 - 2.8e-15) | -11.5 | 1.93E-30 |
| 38 | 6.38E-31 | (7.6e-37 - 5.36e-25) | -9.99 | 1.69E-23 |
| 39 | 4.74E+29 | (7.71e+23 - 2.91e+35) | 10 | 9.42E-24 |
| 40 | 1.68E+28 | (4.85e+22 - 5.84e+33) | 9.99 | 1.76E-23 |
| 41 | 2.90E-35 | (2.22e-42 - 3.79e-28) | -9.51 | 1.86E-21 |
| 42 | 1.48E-32 | (9.44e-39 - 2.32e-26) | -10.1 | 7.53E-24 |
| 43 | 3.54E-26 | (8.31e-31 - 1.51e-21) | -10.8 | 4.46E-27 |
| 44 | 1.77E-33 | (4.05e-40 - 7.72e-27) | -9.67 | 4.15E-22 |
| 45 | 1.22E-26 | (2.07e-31 - 7.25e-22) | -10.6 | 1.91E-26 |
| 46 | 5.80E-29 | (1.37e-34 - 2.45e-23) | -9.84 | 7.71E-23 |
| 47 | 6.17E-44 | (1.81e-53 - 2.1e-34) | -8.88 | 6.40E-19 |
| 48 | 6.43E+32 | (9.81e+25 - 4.21e+39) | 9.43 | 3.97E-21 |
| 49 | 1.57E+23 | (4.62e+18 - 5.33e+27) | 10 | 1.08E-23 |
| 50 | 3.35E-29 | (5.86e-35 - 1.92e-23) | -9.69 | 3.21E-22 |
| 51 | 7.25E-40 | (6.42e-48 - 8.19e-32) | -9.53 | 1.64E-21 |
| 52 | 7.65E-28 | (7.8e-33 - 7.5e-23) | -10.6 | 1.78E-26 |
| 53 | 7.60E+36 | (1.49e+29 - 3.89e+44) | 9.38 | 6.77E-21 |
| 54 | 9.53E+18 | (2.74e+15 - 3.32e+22) | 10.5 | 8.33E-26 |
| 55 | 2.51E+24 | (6.93e+19 - 9.12e+28) | 10.5 | 9.77E-26 |
| 56 | 2.11E+13 | (1.19e+11 - 3.72e+15) | 11.6 | 3.23E-31 |
| 57 | 1.19E-32 | (6.29e-39 - 2.25e-26) | -9.97 | 2.09E-23 |
| 58 | 1.80E-25 | (4.64e-30 - 6.95e-21) | -10.6 | 4.04E-26 |
| 59 | 1.38E+29 | (1.66e+23 - 1.15e+35) | 9.65 | 5.14E-22 |
| 60 | 9.61E+18 | (3.84e+15 - 2.4e+22) | 10.9 | 6.75E-28 |
| 61 | 4.78E-25 | (1.65e-29 - 1.38e-20) | -10.7 | 1.22E-26 |
| 62 | 5.55E+31 | (3.17e+25 - 9.69e+37) | 9.97 | 2.13E-23 |
| 63 | 1.15E+34 | (1.17e+27 - 1.14e+41) | 9.54 | 1.37E-21 |
| 64 | 3.24E-22 | (3.42e-26 - 3.07e-18) | -10.6 | 3.28E-26 |
| 65 | 1.37E+40 | (2.64e+31 - 7.13e+48) | 9.03 | 1.78E-19 |
| 66 | 1.30E-39 | (8.82e-48 - 1.91e-31) | -9.33 | 1.04E-20 |
| 67 | 6.12E+43 | (1.32e+34 - 2.85e+53) | 8.88 | 6.87E-19 |
| 68 | 8.06E-34 | (2.46e-40 - 2.64e-27) | -9.96 | 2.37E-23 |
| 69 | 3.32E+29 | (3.16e+23 - 3.49e+35) | 9.61 | 7.28E-22 |
| 70 | 2.80E-37 | (1.42e-44 - 5.54e-30) | -9.82 | 9.23E-23 |
| 71 | 5.90E+35 | (1.64e+28 - 2.12e+43) | 9.28 | 1.69E-20 |
| 72 | 1.39E-21 | (1.48e-25 - 1.3e-17) | -10.3 | 7.68E-25 |
| 73 | 1.84E-38 | (1.55e-46 - 2.18e-30) | -9.16 | 5.22E-20 |
| 74 | 4.48E-28 | (4.75e-33 - 4.22e-23) | -10.8 | 4.48E-27 |
| 75 | 4.55E-38 | (8.57e-46 - 2.42e-30) | -9.47 | 2.70E-21 |
| 76 | 3.29E+32 | (3.34e+25 - 3.24e+39) | 9.11 | 8.00E-20 |
| 77 | 1.20E-31 | (6.67e-38 - 2.14e-25) | -9.69 | 3.26E-22 |
| 78 | 2.29E-45 | (2.87e-55 - 1.83e-35) | -8.84 | 9.95E-19 |
| 79 | 3.08E-44 | (8.43e-54 - 1.13e-34) | -8.92 | 4.75E-19 |
| 80 | 5.20E+28 | (1.18e+23 - 2.3e+34) | 9.97 | 2.09E-23 |
| 81 | 1.10E+40 | (3.31e+31 - 3.64e+48) | 9.21 | 3.25E-20 |
| 82 | 9.18E+35 | (4.09e+28 - 2.06e+43) | 9.59 | 8.90E-22 |
| 83 | 4.05E-27 | (3.81e-32 - 4.31e-22) | -10.3 | 7.76E-25 |
| 84 | 4.54E-27 | (3.44e-32 - 5.99e-22) | -10.1 | 6.57E-24 |
| 85 | 2.11E-24 | (5.42e-29 - 8.22e-20) | -10.1 | 5.06E-24 |
| 86 | 6.70E+32 | (1.76e+26 - 2.55e+39) | 9.78 | 1.42E-22 |
| 87 | 6.35E-23 | (4.08e-27 - 9.89e-19) | -10.4 | 3.14E-25 |
| 88 | 3.86E+23 | (1.07e+19 - 1.39e+28) | 10.1 | 3.55E-24 |
| 89 | 2.19E+27 | (1.87e+22 - 2.55e+32) | 10.6 | 3.89E-26 |
| 90 | 6.13E-32 | (2.58e-38 - 1.45e-25) | -9.6 | 8.36E-22 |
| 91 | 3.64E+24 | (5.68e+19 - 2.33e+29) | 10 | 1.31E-23 |
| 92 | 1.11E+22 | (1.34e+18 - 9.19e+25) | 11 | 2.88E-28 |
| 93 | 8.32E+27 | (3.05e+22 - 2.27e+33) | 10.1 | 7.77E-24 |
| 94 | 2.30E-31 | (1.77e-37 - 2.99e-25) | -9.82 | 9.02E-23 |
| 95 | 1.58E+25 | (2.71e+20 - 9.26e+29) | 10.4 | 3.69E-25 |
| 96 | 3.27E-31 | (2.27e-37 - 4.72e-25) | -9.7 | 2.97E-22 |
| 97 | 2.82E+45 | (4.87e+35 - 1.63e+55) | 9.12 | 7.20E-20 |
| 98 | 4.25E+26 | (3.32e+21 - 5.43e+31) | 10.2 | 1.62E-24 |
| 99 | 1.10E-20 | (2.47e-24 - 4.86e-17) | -10.7 | 7.59E-27 |
| 100 | 1.63E+29 | (3e+23 - 8.83e+34) | 9.98 | 1.80E-23 |

A summary of the results from the univariate cox models for each bottleneck feature including hazard ratios (HR) and 95% confidence intervals (CI). Results are based on the bottleneck features from the best run of L_RSC_ as determined by log-rank *P*-value as presented in Table 2.

**Table S2. Methylation features consistently identified in ten runs of L_RSC_ that are significant for prognosis.**

| Methylation Feature | Log-rank *P*-value |
| --- | --- |
| *PON2* | 1.05E-06 |
| *SOCS2* | 2.44E-05 |
| *CAPS2* | 3.33E-05 |
| *RICTOR* | 3.50E-05 |
| *SPRY2* | 4.05E-05 |
| *GAR1* | 9.09E-05 |
| *ARHGAP5* | 1.02E-04 |
| *ARHGAP5-AS1* | 1.02E-04 |
| *PRR13* | 1.08E-04 |
| *GMFB* | 1.27E-04 |
| *AZI2* | 1.30E-04 |
| *AMY2B* | 1.57E-04 |
| *ALG10B* | 1.97E-04 |
| *OMG* | 2.01E-04 |
| *AKR1C8P* | 2.16E-04 |
| *NIPBL* | 2.22E-04 |
| *PDCL3* | 2.22E-04 |
| *KLHL8* | 2.51E-04 |
| *CCDC18* | 2.55E-04 |
| *TMED5* | 2.55E-04 |
| *TMEM260* | 2.63E-04 |
| *SPATA7* | 2.75E-04 |
| *IFI27L1* | 2.96E-04 |
| *DDX24* | 2.96E-04 |
| *PAK2* | 2.98E-04 |
| *CCDC91* | 3.00E-04 |
| *RAD17* | 3.10E-04 |
| *TAF9* | 3.25E-04 |
| *CXorf21* | 3.29E-04 |
| *CETN3* | 3.31E-04 |
| *AP1S1* | 3.56E-04 |
| *ZNF680* | 3.77E-04 |
| *THRAP3* | 3.78E-04 |
| *MIR933* | 3.83E-04 |
| *ATF2* | 3.83E-04 |
| *KAT2B* | 3.98E-04 |
| *SSMEM1* | 4.27E-04 |
| *TMEM106B* | 4.28E-04 |
| *KAT6B* | 4.39E-04 |
| *CHCHD3* | 4.61E-04 |
| *IFT80* | 5.26E-04 |
| *LPXN* | 5.73E-04 |
| *IMMP1L* | 5.78E-04 |
| *ELP4* | 5.78E-04 |
| *TLK1* | 5.94E-04 |
| *HELB* | 6.03E-04 |
| *TTC17* | 6.12E-04 |
| *ARPC5* | 6.12E-04 |
| *ZNF688* | 6.44E-04 |
| *ZFP91* | 6.48E-04 |
| *SP1* | 6.64E-04 |
| *COPB1* | 7.15E-04 |
| *VAPB* | 8.12E-04 |
| *LOC550643* | 8.16E-04 |
| *TWF1* | 8.33E-04 |
| *EIF1* | 8.49E-04 |
| *AGO3* | 8.56E-04 |
| *SPCS3* | 8.61E-04 |
| *KIF14* | 8.84E-04 |
| *TRAPPC13* | 9.04E-04 |
| *TRIM23* | 9.04E-04 |
| *C1orf122* | 9.26E-04 |
| *YRDC* | 9.26E-04 |
| *CEP290* | 1.00E-03 |
| *TMTC3* | 1.00E-03 |
| *RHEBL1* | 1.06E-03 |
| *EFCAB7* | 1.12E-03 |
| *ITGB3BP* | 1.12E-03 |
| *C1orf109* | 1.12E-03 |
| *ATF6* | 1.14E-03 |
| *B3GALT2* | 1.29E-03 |
| *USF1* | 1.35E-03 |
| *PCNP* | 1.44E-03 |
| *IKZF2* | 1.45E-03 |
| *LONP1* | 1.48E-03 |
| *CATSPERD* | 1.48E-03 |
| *SLC25A44* | 1.50E-03 |
| *PMF1* | 1.50E-03 |
| *CBWD1* | 1.51E-03 |
| *RMI2* | 1.63E-03 |
| *NGLY1* | 1.64E-03 |
| *G2E3* | 1.68E-03 |
| *MPHOSPH9* | 1.81E-03 |
| *TMTC4* | 1.90E-03 |
| *ARHGEF18* | 1.93E-03 |
| *TUBAL3* | 1.98E-03 |
| *OTUD6B* | 2.09E-03 |
| *MSMO1* | 2.27E-03 |
| *MIR1296* | 2.42E-03 |
| *TMEM104* | 2.58E-03 |
| *NAT9* | 2.58E-03 |
| *ZNF780B* | 2.66E-03 |
| *MAGI3* | 2.75E-03 |
| *IL1R1* | 2.82E-03 |
| *MRPS5* | 2.85E-03 |
| *PLEKHA3* | 2.86E-03 |
| *ADAMTS6* | 2.90E-03 |
| *NSUN2* | 3.12E-03 |
| *SRD5A1* | 3.12E-03 |
| *SNHG4* | 3.13E-03 |
| *GDPD1* | 3.19E-03 |
| *C12orf73* | 3.19E-03 |
| *CDCA8* | 3.23E-03 |
| *LUC7L2* | 3.29E-03 |
| *KIF1B* | 3.33E-03 |
| *CTR9* | 3.33E-03 |
| *EGLN1* | 3.42E-03 |
| *EIF2A* | 3.50E-03 |
| *SERP1* | 3.50E-03 |
| *OSGEP* | 3.53E-03 |
| *APEX1* | 3.53E-03 |
| *SYDE2* | 3.56E-03 |
| *ZKSCAN5* | 3.58E-03 |
| *EXOC5* | 3.59E-03 |
| *AP5M1* | 3.59E-03 |
| *MTMR10* | 3.64E-03 |
| *SLC3A2* | 3.73E-03 |
| *NUP153* | 3.87E-03 |
| *SPDYA* | 3.90E-03 |
| *CTNNAL1* | 4.08E-03 |
| *RNPC3* | 4.12E-03 |
| *FUBP1* | 4.18E-03 |
| *SLC8B1* | 4.31E-03 |
| *PHF14* | 4.32E-03 |
| *PDS5A* | 4.36E-03 |
| *BDNF-AS* | 4.39E-03 |
| *ASB3* | 4.47E-03 |
| *ARRDC3* | 4.72E-03 |
| *LOC100129716* | 4.72E-03 |
| *LMO4* | 4.83E-03 |
| *CERT1* | 4.99E-03 |
| *POLK* | 4.99E-03 |
| *SELENOF* | 5.06E-03 |
| *HS2ST1* | 5.06E-03 |
| *MIRLET7I* | 5.20E-03 |
| *MSANTD2* | 5.64E-03 |
| *PLEKHA8P1* | 5.73E-03 |
| *MMAA* | 5.83E-03 |
| *CENPC* | 5.87E-03 |
| *CWF19L2* | 6.20E-03 |
| *WDR89* | 6.28E-03 |
| *LSM5* | 6.30E-03 |
| *TRMT13* | 6.40E-03 |
| *SASS6* | 6.40E-03 |
| *NT5C3A* | 6.64E-03 |
| *CFAP70* | 6.70E-03 |
| *MGC87042* | 7.22E-03 |
| *LOC283914* | 7.23E-03 |
| *SPEF1* | 7.37E-03 |
| *TPD52L1* | 7.69E-03 |
| *ZNF251* | 7.80E-03 |
| *EFNA3* | 7.89E-03 |
| *LOC552889* | 7.94E-03 |
| *MFSD14A* | 8.19E-03 |
| *ATG10* | 8.21E-03 |
| *MTF1* | 8.36E-03 |
| *CFAP298* | 8.94E-03 |
| *BCO2* | 9.20E-03 |
| *GARS1* | 9.44E-03 |
| *TTLL3* | 9.49E-03 |
| *FKBP7* | 9.66E-03 |
| *C6orf120* | 1.00E-02 |
| *WDR27* | 1.00E-02 |
| *TAS2R14* | 1.01E-02 |
| *AP5Z1* | 1.11E-02 |
| *GPX8* | 1.25E-02 |
| *HERC1* | 1.28E-02 |
| *MIR1539* | 1.35E-02 |
| *LETMD1* | 1.35E-02 |
| *ASNSD1* | 1.36E-02 |
| *CC2D2B* | 1.37E-02 |
| *CAV2* | 1.40E-02 |
| *AMMECR1* | 1.46E-02 |
| *CYP21A2* | 1.52E-02 |
| *TRIP12* | 1.54E-02 |
| *FBXO36* | 1.54E-02 |
| *C18orf32* | 1.58E-02 |
| *GP6* | 1.61E-02 |
| *POU2F1* | 1.69E-02 |
| *KDM3A* | 1.70E-02 |
| *DHRS4L2* | 1.77E-02 |
| *CIR1* | 1.80E-02 |
| *SCRN3* | 1.80E-02 |
| *ARHGEF35* | 1.81E-02 |
| *USP46* | 1.87E-02 |
| *RTL9* | 1.87E-02 |
| *SLC17A3* | 1.90E-02 |
| *BCL10* | 2.01E-02 |
| *VXN* | 2.04E-02 |
| *PPP4C* | 2.04E-02 |
| *NEK4* | 2.07E-02 |
| *KIAA0586* | 2.08E-02 |
| *TIMM9* | 2.08E-02 |
| *MBD1* | 2.16E-02 |
| *UHRF1BP1L* | 2.19E-02 |
| *MAN2B1* | 2.23E-02 |
| *SNORD2* | 2.24E-02 |
| *C7* | 2.26E-02 |
| *POP4* | 2.36E-02 |
| *ABCD2* | 2.40E-02 |
| *ERO1B* | 2.40E-02 |
| *MIR591* | 2.41E-02 |
| *SNX13* | 2.50E-02 |
| *FBXO4* | 2.51E-02 |
| *DYNLT3* | 2.52E-02 |
| *SHBG* | 2.54E-02 |
| *SIGLEC15* | 2.74E-02 |
| *RNF11* | 2.84E-02 |
| *ERLEC1* | 2.85E-02 |
| *C12orf43* | 2.86E-02 |
| *PPARGC1B* | 2.86E-02 |
| *BICD2* | 2.87E-02 |
| *RPS15AP10* | 2.99E-02 |
| *NAT8* | 3.22E-02 |
| *AKR1B10* | 3.29E-02 |
| *ICAM4* | 3.42E-02 |
| *GFRA3* | 3.58E-02 |
| *ZSCAN32* | 3.68E-02 |
| *ZNF174* | 3.68E-02 |
| *UBA5* | 3.89E-02 |
| *PKN2* | 4.02E-02 |
| *HSPA6* | 4.05E-02 |
| *PREPL* | 4.13E-02 |
| *CAMKMT* | 4.13E-02 |
| *KIAA1522* | 4.51E-02 |
| *DMXL1* | 4.65E-02 |
| *SF3A2* | 4.82E-02 |
| *LEXM* | 4.89E-02 |
| *DGKE* | 4.92E-02 |
| *CTDP1* | 4.97E-02 |
| *SNRNP200* | 4.98E-02 |
| *TYW1B* | 5.00E-02 |
| *SBDSP1* | 5.00E-02 |

A list of the 233 overlapping methylation features identified by the hybrid model that were significant and consistently identified in ten runs of L_RSC_. Results show their prognostic value using a log-rank test with a median split.
